# Numerical Simulation of Streaming Discharge Characteristics of Free Metal Particles in SF_6_/CF_4_ Gas Mixtures Under Highly Heterogeneous Electric Field

**DOI:** 10.3390/s25133847

**Published:** 2025-06-20

**Authors:** Bing Qi, Hui Wang, Chang Liu, Fuyou Teng, Daoxin Yu, Yuxuan Liang, Feihu Wang

**Affiliations:** Key Laboratory of Modern Power System Simulation and Control and Renewable Energy Technology, Ministry of Education, School of Electrical Engineering, Northeast Electric Power University, Jilin City 132012, China; qibing@neepu.edu.cn (B.Q.);

**Keywords:** SF_6_/CF_4_, positive stream, free metal particle, suspension potential

## Abstract

Compared to pure SF_6_ gas, the SF_6_/CF_4_ gas mixture exhibits certain advantages in reducing greenhouse effects, lowering the liquefaction temperature, and decreasing the sensitivity to non-uniform electric fields, demonstrating significant application potential in high-voltage electrical equipment. This study employs a two-dimensional plasma fluid model to investigate the partial discharge phenomena induced by free metallic particles in SF_6_/CF_4_ gas mixtures, analyzing the spatiotemporal evolution characteristics of key parameters, such as the charged particle density and axial electric field, under different mixing ratios. The simulation results show that there are two kinds of positive stream discharge phenomena, “continuous and decaying”, when the gas mixture ratio is 90%CF_4_-10%SF_6_ and 40%CF_4_-60%SF_6_. The proportion of CF_4_ in the gas mixture will affect the spatial distribution of charged particles and the production and disappearance of electrons. When the proportion of CF_4_ is 90%, the content of positive ions in the discharge channel is the highest, and the electric field formed by the positive space charge of CF_4_^+^ in the stream head promotes the continuous propagation of the stream. As the concentration of CF_4_ decreases, the main ionization reaction at the stream head shifts from CF_4_ to SF_6_, and a negative space charge region dominated by SF_6_^−^ particles is also formed near the stream head, changing the electric field distribution near the flow head. The adhesion reaction rate is greater than the ionization reaction rate, resulting in the disappearance of electrons greater than the production, and the stream phenomenon tends to decay. These simulation results are helpful to understand the dynamic process of positive stream discharge induced by free metal particles in SF_6_/CF_4_ gas mixtures, and they provide a theoretical basis for better solutions to equipment damage caused by partial discharge.

## 1. Introduction

SF_6_ gas, known for its exceptional electrical insulation and arc-quenching properties, has been extensively utilized in high-voltage equipment such as gas-insulated switchgear (GIS), gas-insulated transmission lines (GILs), and high-voltage circuit breakers. However, in recent years, the global power system has undergone significant transformations, with a growing emphasis on the adoption of environmentally friendly materials. Given that SF_6_ possesses an extremely high global warming potential (GWP), approximately 23,900 times greater than that of CO_2_ [1,2], the prevailing trend is to minimize or eliminate its usage. In addition, the tendency of SF_6_ to liquefy at low temperatures imposes constraints on its practical application in high-latitude regions. To date, no gas has been identified that can fully substitute for SF_6_ in terms of both its insulation and arc-quenching performance. The most effective strategy to address this issue is to reduce its usage within power systems. Employing SF_6_-based gas mixtures represents a viable solution [3,4]. Commonly, SF_6_ is blended with N_2_, CO_2_, CF_4_ or certain rare gases. Notably, CF_4_ exhibits a GWP value that is only one-third that of SF_6_, with a liquefaction temperature under standard atmospheric pressure as low as −128 °C. Additionally, CF_4_ demonstrates strong electronegativity, a superior arc-quenching capability, and favorable thermodynamic properties [5]. Research indicates that when CF_4_ and SF_6_ are mixed in a specific ratio, the resulting mixture exhibits an elevated liquefaction temperature while preserving excellent insulation properties [6]. To date, the SF_6_/CF_4_ gas mixture has been extensively utilized as an insulating medium for high-voltage circuit breakers in extremely cold regions [7,8,9].

In SF_6_-based, high-voltage, sealed electrical equipment, a significant proportion of insulation failures are associated with foreign substances, such as metal particles. These metal particles primarily result from mechanical impacts, vibrations, and friction during the processes of manufacturing, installation, transportation, and operation of the equipment. At accident sites, millimeter-sized metal particles are frequently observed adhering to the equipment surfaces, along with a limited number of micrometer-sized or smaller metal dust particles [10]. Such particles may either be in direct contact with components or remain in a suspended state, leading to partial discharge phenomena. Partial discharge progressively degrades the insulation of equipment, and under severe conditions, it may evolve into a flashover, ultimately compromising the insulation performance of high-voltage equipment [11]. Consequently, conducting in-depth research on the partial discharge phenomenon induced by metal particles is crucial for comprehending the progression of partial discharge, refining diagnostic and mitigation strategies, and improving the safety and reliability of power equipment. To achieve a more comprehensive understanding of the occurrence and evolution mechanisms of partial discharge induced by metal particles in SF_6,_ researchers have carried out a series of systematic experiments. These experiments primarily investigated the behavior of free metal particles under the following conditions: resting on insulating material surfaces, adhering to metal shell surfaces, or existing in a suspended or freely moving state [12,13,14]. When free metal particles make contact with high-voltage electrodes or grounding terminals, they form an equipotential structure, which facilitates the simplification of simulation processes and enhances the stability of the discharge phenomenon. However, when metal particles are in a suspended state, their potential varies in accordance with the spatial electric potential. The metal particles and the surrounding gas medium collectively form a capacitive system. In terms of millimeter-scale metal particles, they typically can only accommodate charge quantities on the order of picocoulombs [15]. The transient charging and discharging process induced by partial discharge ceases within an extremely short duration due to the minuscule charge transfer and relaxation times in the sub-nanosecond to nanosecond range. This characteristic imparts ultrafast time-response features to the partial discharge signals generated by free metal particles. Conventional detection techniques often struggle to fully capture these transient processes owing to bandwidth constraints. Furthermore, the weak signal amplitudes pose significant challenges in effectively differentiating them from background noise, which has hindered detailed investigations into the partial discharge characteristics and evolution mechanisms of millimeter-scale free metal particles. Additionally, partial discharge arises from the synergistic interactions of multiple physical processes, such as charge generation and transport, charge energy transfer, molecular excitation, and ionization. These processes are interdependent and mutually restrictive; thus, they increase the complexity of the system and render it difficult to characterize using macroscopic parameters obtained experimentally. Consequently, experimental approaches exhibit substantial limitations in elucidating the underlying discharge mechanisms.

Finite element simulation represents an effective approach for investigating the discharge process in SF_6_ mixed gas. The fluid model, which is grounded in transport equations, is frequently employed. Based on the local field approximation assumption, this model can accurately capture the microscopic motion dynamics of charged particles with adequate precision [16,17,18]. The reasonable adjustment of the parameters informed by experimental data and Boltzmann equation results establishes a solid foundation for simulation studies utilizing the fluid model [19,20,21,22]. However, fluid models are incapable of capturing the variations in specific substances and also struggle to adequately represent surface reactions on dielectrics and electrodes. Plasma fluid models that incorporate specific particle reactions [23] have gained increasing application in gas discharge simulation studies in recent years. These models can capture the dynamics of specific particles within complex gas reactions [24,25,26,27] and more effectively address surface reactions at boundaries [28]. Lijun Wang [29] developed a plasma fluid model for discharge in non-uniform fields using COMSOL Multiphysics. Through numerical simulations, the spatiotemporal evolution of the electron density, positive ion density, negative ion density, and electric field intensity during the negative streamer discharge of SF_6_/N_2_ mixed gas was investigated. The effects of varying concentration ratios on the streamer development were examined; however, the fundamental mechanisms underlying their influence on the streamer discharge were not fully elucidated. Bin Luo [25] conducted a simulation of the positive streamer discharge process. By modulating the applied voltage, the spatiotemporal evolution of the streamer propagation and attenuation was systematically analyzed. The accumulation of negative charges in the spatial domain suppressed the ionization of SF_6_ and N_2_ at the streamer head, consequently influencing the streamer’s propagation characteristics. Zhen Li [15] also employed the plasma fluid model, with SF_6_/N_2_ mixed gas as the research medium, to analyze the propagation characteristics of positive streamers under rod–plate electrodes. Additionally, the study investigated the effects of varying the mixing ratios, pressures, and temperatures on the streamer thickness.

Currently, the investigation into the streamer discharge process of SF_6_/N_2_ mixed gas has reached a relatively mature stage, whereas research on the discharge phenomena of SF_6_/CF_4_ mixed gas remains limited. Further in-depth studies are urgently required in this field to comprehensively elucidate the associated discharge characteristics and underlying mechanisms. Dengming Xiao et al. [30,31,32] performed a detailed computational analysis of the electronic transport parameters at room temperature. The research team, led by Jia Zhang and Hu Zhao [33], derived the discharge parameters for 50%/50% SF_6_/CF_4_ mixed gas at one atmosphere pressure using two approximate methods to solve the Boltzmann equation. All prior studies on SF_6_/CF_4_ mixed gas have been conducted at the macroscopic level, without considering the impact of chemical reactions on discharge phenomena. In this paper, suspended free metal particles are investigated as the research subject, and the plasma fluid model is employed to explore the streamer phenomenon induced in the discharge space. Additionally, the spatiotemporal evolution characteristics of key parameters, such as the charged particle density and axial electric field strength, are analyzed under different mixing ratios.

## 2. Discharge Model and Parameters

### 2.1. Mathematical Model

The fluid model treats the plasma generated by partial discharge as a continuous medium consisting of electrons, ions, and neutral particles, and it characterizes these species in terms of their concentration, average velocity, and average energy. The continuity equation, momentum equation, and energy equation derived from the Boltzmann equation are utilized to determine the parameters of these particles. The Poisson equation is integrated with the previously mentioned equations to determine the spatial arrangement of the electric potential.

The continuity equation can be expressed as follows:

(1) $$\frac{\partial n_{e,i}}{\partial t}+\nabla \cdot \Gamma_{e,i}=S_{e,i}$$

In this equation, *e* and *i* denote electrons and ions, respectively; *t* represents time; $n_{e,i}$ signifies the particle number density; $\Gamma_{e,i}$ corresponds to the particle flux derived from the transport–diffusion equation; and $S_{e,i}$ indicates the particle source term.

The mathematical formulation of the transport–diffusion equation is presented as follows:

(2) $$\Gamma_{e,i}=\mp q_{e,i}n_{e,i}\mu_{e,i}E-D_{e,i}\nabla n_{e,i}$$

In the formula, $q_{e,i}$ denotes the charge of the charged particle, $\mu_{e,i}$ represents its mobility, E corresponds to the electric field strength, and $D_{e,i}$ signifies the diffusion coefficient of the charged particle.

The source term ($S_{e,i}$) expression of Equation (1) is as follows:

(3) $$S_{e,i}=\sum_{j=1}^{M}x_{j}k_{j}N_{n}n_{e}$$

Here, *M* denotes the total number of electrons or ions; $x_{j}$ represents the mole fraction of the *j*-th charged particle; $k_{j}$ signifies the rate coefficient of the *j*-th reaction; and $N_{n}$ indicates the density of neutral particles.

The rate coefficient can be determined by evaluating the following integral using the cross-section data:

(4) $$k_{j}=\gamma \int_{0}^{\infty }\varepsilon \sigma_{k}(\varepsilon )f(\varepsilon )d\varepsilon$$

In the formula, $\gamma ={\left(2E/m_{e}\right)}^{1/2}$, ε denotes the electron energy, $\sigma_{k}$ represents the collision cross section, f(ε) corresponds to the electron energy distribution function (EEDF), and *E* is the kinetic energy of the electron, where $m_{e}$ is the electron mass and *e* denotes the electron. Under these conditions, the electron temperature distribution function is assumed to be based on the two-term approximation of the Boltzmann equation.

The determination of the electron temperature necessitates solving the equation for the conservation of electron energy:

(5) $$\frac{\partial n_{\varepsilon }}{\partial t}+\nabla \cdot \Gamma_{\varepsilon }=S_{T_{e}}$$

In the formula, $n_{\varepsilon }=n_{e}\bar{\varepsilon }$ denotes the electron energy density, $\bar{\varepsilon }$ represents the electron energy, and $\Gamma_{\varepsilon }$ represents the electron energy flux density, which can be written as follows:

(6) $$\Gamma_{\varepsilon }=\frac{5}{3}n_{e}\mu_{e}E-\frac{5}{3}D_{E}\nabla n_{e}$$

$S_{T_{e}}$ represents the source of electronic energy and is mathematically expressed as follows:

(7) $$S_{T_{e}}=-e\Gamma_{e}E-\sum_{j}\Delta E_{j}R_{inel.j}-3\frac{m_{e}}{M}k_{b}n_{e}\gamma_{en}(T_{e}-T_{g})$$

In the formula, $\Delta E_{j}$ and $R_{inel.j}$ denote the loss of energy due to elastic collisions and the corresponding reaction rate, respectively. Here, $m_{e}$ and *M* represent the masses of electrons and neutral gas particles, $k_{b}$ represents the Boltzmann constant, $\gamma_{en}$ denotes the collision frequency between electrons and neutral particles, $T_{e}$ corresponds to the electron temperature, and $T_{g}$ refers to the gas temperature. The $S_{T_{e}}$ term is composed of three components: Joule heating induced by the electric field, energy loss resulting from inelastic collisions between electrons and heavy particles, and the kinetic energy transferred to heavy ions in elastic collisions between electrons and heavy particles.

The spatial potential distribution is determined by the superposition of the externally applied electric field and the electric field generated by space charges, and it can be mathematically described using Poisson’s equation:

(8) $$\nabla^{2}\phi =-\frac{\rho }{\varepsilon_{0}\varepsilon_{r}}$$

Here, ϕ denotes the spatial electric potential, $\varepsilon_{0}$ represents the vacuum permittivity, and $\varepsilon_{r}$ indicates the relative permittivity. The symbol ρ refers to the spatial charge density, which can be mathematically expressed as follows:

(9) $$\rho =q(\sum_{k=1}^{N}Z_{k}n_{k}-n_{e})$$

Here, *ρ* denotes the spatial charge density, *q* signifies the elementary charge, $Z_{k}$ indicates the number of charges carried by the *k*-th positive particle, and $n_{k}$ refers to the density of the *k*-th positive particle.

### 2.2. Reaction Equations and Collision Cross Sections

To improve the accuracy of the simulation results, the fluid dynamics model incorporated a comprehensive analysis of the primary reaction processes of SF_6_ and CF_4_. The specific reactions are detailed in Table 1 [1], encompassing ionization reactions (R9, R18), attachment reactions (R1–R3, R19–R12), excitation reactions (R5–R8, R14–R17), and elastic collision reactions (R4, R13). These reactions provide an effective description of the generation, consumption, and mutual transformation of positively and negatively charged particles within the discharge channel. In the discharge channel, the predominant positive charge carriers are SF_6_^+^ and CF_4_^+^ ions, but negative charge carriers, including SF_6_^+^, SF_5_^−^, SF_4_^−^, F_2_^−^ anions, and free electrons, are also present.

**Figure 1:**
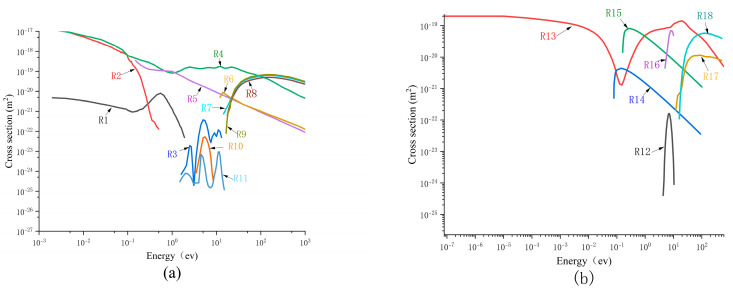
Collision cross section: (**a**) SF_6_; (**b**) CF_4_.

Figure 1 illustrates the collision cross-section data for SF_6_ and CF_4_. The collision cross section serves as a critical parameter for quantifying the interaction strength between gas molecules and electrons or ions during collisions, which directly affect the particle dynamics behavior in the discharge process. The collision cross-section data presented in the figure are sourced from Lxcat [34], a reputable database that provides a robust physical parameter foundation for simulations, thereby ensuring the precision and validity of the simulation results.

### 2.3. Boundary Conditions and Solver Configuration

**Figure 2:**
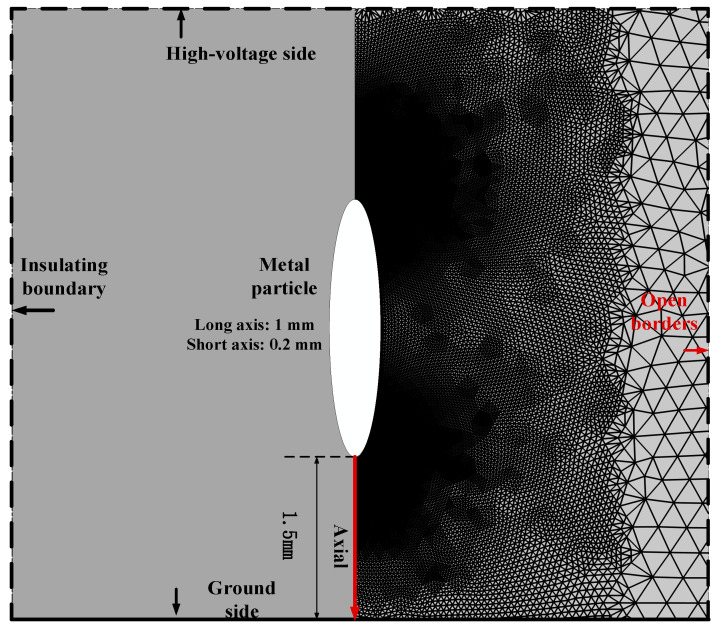
Simulation geometric model.

Figure 2 presents the geometric model employed in the simulation, with a two-dimensional domain of the dimensions 10 mm × 40 mm. The metal particles are represented as ellipses, characterized by a major axis of 1 mm and a minor axis of 0.2 mm, located at a distance of 1.5 mm from the lower electrode. In COMSOL Multiphysics, a constant voltage of 27.5 kV is applied to the high-voltage side via metal contact. The lower boundary is grounded, the left boundary is insulated, and the right boundary is defined as open space [35]. To account for possible charge accumulation at the insulating boundary, dielectric contact is employed to define the charge accumulation boundary condition. The metal particles are assigned a floating potential. As these particles exchange charges with the surrounding plasma, their potentials evolve over time. By coupling the Current Module with the Plasma Module, the currents flowing into and out of the particle boundaries can be computed, enabling the determination of the particles’ floating potential.

In COMSOL Multiphysics the finite element method (FEM) is utilized to solve partial differential equations (PDEs) and transform them into discrete linear algebraic equations. This study employs a two-dimensional plasma model that incorporates multi-physics coupling and exhibits significant nonlinear characteristics. An automatic solver tailored for highly nonlinear problems is adopted, ensuring an improved convergence performance. To improve the simulation accuracy, the mesh near the metal particles is refined, as illustrated in Figure 2, with the smallest mesh element size being 2.5 × 10^−3^ mm. Furthermore, to resolve the issue of calculation failure caused by large residuals, the maximum time step is set to 10^−4^ ns, the number of iterations is increased to 25, and the tolerance limit is adjusted to 1. Although these settings will lead to an extension of the simulation time, to a certain extent, they ensure that the simulation results have good convergence and tend to be stable. Photoionization represents the most critical mechanism for generating seed electrons in the non-local region of the positive streamer head. However, when employing COMSOL to simulate positive streamer discharge phenomena, the photoionization effect cannot be adequately accounted for through chemical kinetics equations alone. To resolve this limitation, the pre-ionization method can be utilized as an alternative to approximate the photoionization effect [36]. Specifically, it is assumed that at the onset of discharge, a spatial distribution of seed electrons with a density of 10^15^ m^−3^ exists.

## 3. Result Analysis

**Figure 3:**
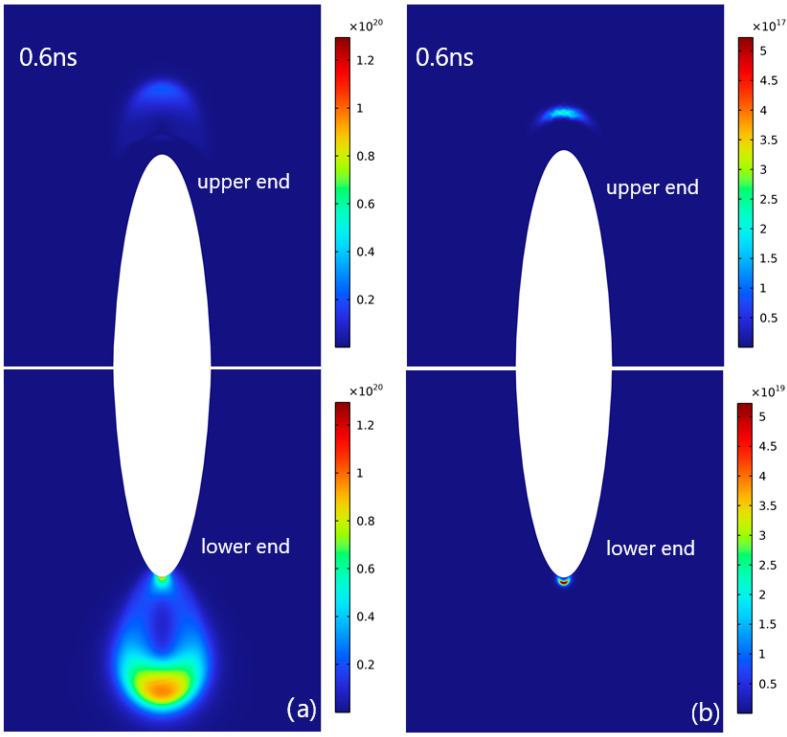
Electron density distributions during “double-headed” discharge around metal particles for different gas ratios. Electron density is measured in m^−3^: (**a**) 90% CF_4_-10% SF_6_; (**b**) 40%CF_4_-60%SF_6_.

The spatial electron density distributions for two distinct concentrations of SF_6_/CF_4_ mixed gas at 0.6 ns are illustrated in Figure 3. Metal particles are suspended within the discharge space, and simultaneous discharges occur at both the upper and lower tips, a phenomenon known as the “double-head” discharge mode in certain research studies [35]. Metal particles can be approximated as equipotential bodies. When placed between a high-voltage electrode and grounded electrode, the particle exhibits a lower potential relative to the high-voltage side and a higher potential relative to the grounded electrode. The discharge phenomenon at the upper end manifests as a negative streamer, whereas that at the lower end takes the form of a positive streamer. In order to clearly demonstrate the discharge process, distinct electron density color scales are employed for the upper and lower regions and are positioned on the corresponding sides. Given that the electron density at the lower end of the discharge region is 1–2 orders of magnitude higher than that at the upper end, the discharge process is significantly more intense. Consequently, this study primarily investigates the underlying mechanisms of the discharge at the lower end of the metal particles.

**Figure 4:**
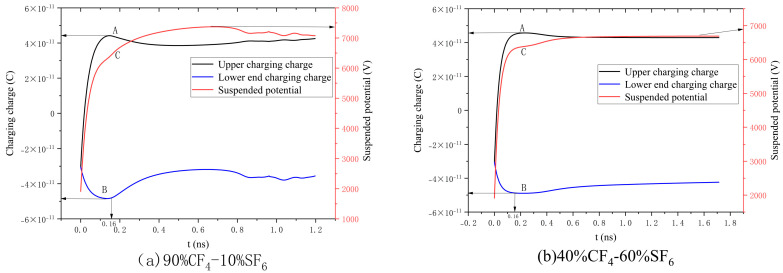
Charging charge and suspension potential on metal particle surfaces during discharge of varying gas mixtures. A: The charging charge at the upper part, B: the charging charge at the lower part, and C: the surface potential of the suspension.

Figure 4a illustrates the variations in the charging charge and surface floating potential during the discharge process for an SF_6_/CF_4_ gas mixture ratio of 10%/90%. Specifically, A denotes the charging charge at the upper end, B corresponds to the charging charge at the lower end, and C indicates the surface floating potential. After the discharge process begins, under the influence of the external electric field, positive and negative charges surrounding the metal particles undergo directed movement. This results in the accumulation of positive charges on the upper surface of the particles and negative charges on the lower surface. The charging process is accomplished within an extremely short time frame at the initiation of the discharge. During this period, the potential of the metal particles varies dynamically as a function of the accumulated positive and negative charges. Notably, throughout the charging phase, positive charges dominate the distribution, and the floating potential increases sharply within 0.16 ns. At this time point (0.16 ns), a transition in the dynamic behavior of both the charging process and the floating potential is observed, after which both parameters progressively approach their respective steady-state values. Figure 4b illustrates the variations in the charging charge and suspended potential for an SF_6_/CF_4_ gas mixture ratio of 60%/40%. In comparison with the 90% CF_4_-10% SF_6_ gas mixture, the suspended potential and charging charge reach a certain level and exhibit significant stability. Nevertheless, the overall trends of variation in both the charging charge and suspended potential remain consistent. This study does not explore in depth the specific mechanism by which differences in the mixing ratio influence the charging and discharging of particles, as this involves a complex coupling process across multiple physical fields, which lies beyond the scope of the current investigation.

**Figure 5:**
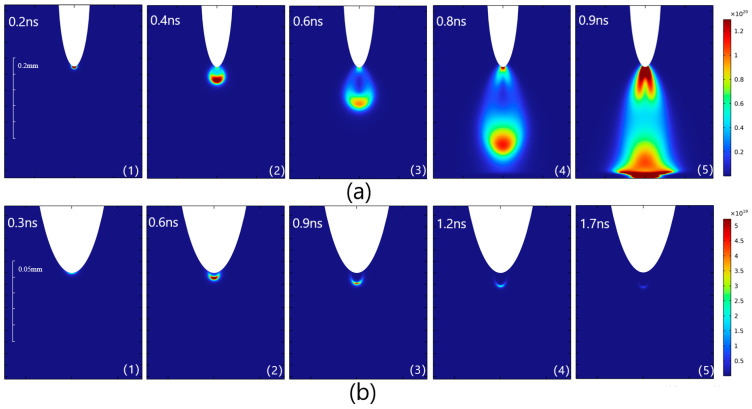
Spatial electron distributions during discharge of different gas mixtures: (**a**) 90%CF_4_-10%SF_6_; (**b**) 40%CF_4_-60%SF_6_.

To investigate the impact of the SF_6_/CF_4_ gas ratio on the streamer propagation, simulations were conducted for 90% CF_4_-10% SF_6_ and 40% CF_4_-60% SF_6_ mixtures. Figure 5 presents the spatial electron density distributions under these two gas compositions. When the CF_4_ concentration was 90%, as depicted in Figure 5a, the streamer exhibited continuous propagation. After detaching from the tip, the streamer expanded both radially and axially, forming a “water drop”-like shape. The propagation speed of the streamer was estimated to range from 6.7 × 10^5^ m/s to 1.6 × 10^6^ m/s, based on the distance it extended toward the grounding electrode per unit time, and the streamer reached the ground electrode within 0.9 ns. This phenomenon is referred to as the “continuous propagation” regime in this study. The continuous transmission speed is comparable to the streamer propagation speed of 1 × 10^6^ m/s~1.2 × 10^6^ m/s observed in the 20% SF_6_/80% N_2_ gas mixture, as reported in Luo et al. [25]. However, this value is significantly higher than the propagation speed of pure SF_6_ reported in Reference [20], which ranges from 7 × 10^5^ m/s to 8.25 × 10^5^ m/s.

When the CF_4_ concentration was 40%, the streamer took on a “crescent” shape, with a propagation speed ranging from approximately 5 × 10^4^ m/s to 6.4 × 10^4^ m/s. The streamer dissipated within 1.7 ns under these conditions. This phenomenon is referred to as the “attenuation propagation” regime in this study, with the maximum streamer propagation distance being 0.07 mm. Under strictly controlled simulation conditions of constant temperature and pressure, the differences between the two discharge modes can be attributed to variations in the gas mixing ratio. A quantitative analysis of the propagation speed reveals that the peak speed in the continuous propagation regime is approximately two orders of magnitude higher than that in the attenuation regime. These results suggest that the CF_4_ concentration significantly influences the streamer propagation speed.

**Figure 6:**
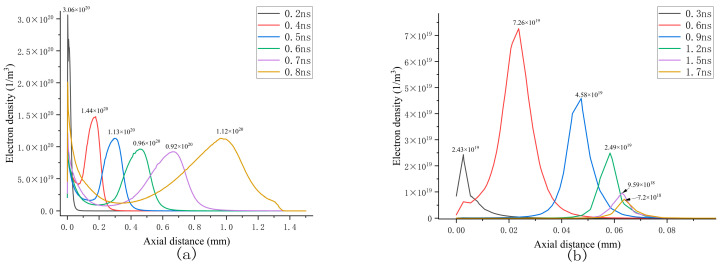
Axial electron distributions of continuous propagation and attenuation propagation: (**a**) continuous transmission situation; (**b**) attenuation propagation situation.

Figure 6a illustrates the axial electron density in the continuous propagation scenario, with an order of magnitude of 10^20^ m^−3^. Starting from the initial state (t = 0 ns), the electron region detaches from the particle tip, forming a localized electron density peak. At 0.2 ns, due to the streamer head being near the particle, there exists a significant distorted electric field, which enhances the ionization rate of the gas, and the peak electron density increases to 3.06 × 10^20^ m^−3^. When the streamer moves away from the particle tip, the effect brought on by the distorted electric field weakens. As the streamer approaches the grounded electrode, the electric field increases, which causes the peak electron density to increase again. Overall, the peak electron density shows a two-stage evolution characteristic of first decaying and then increasing. The axial electric field distribution depicted in Figure 10a reveals that the applied electric field induces distortion near the tip, leading to the convergence of secondary electron avalanches and driving the electron region toward the grounded electrode. As the streamer moves away from the particle tip, the self-consistent electric field generated by the space charge gradually becomes dominant. This field continuously facilitates the convergence of secondary electron avalanches, sustaining the propagation of the streamer. The evolution of the spatial electron density is illustrated in Figure 5(a1–a5). During this dynamic process, the driving effect of the applied electric field exhibits a progressive attenuation trend. Figure 6b illustrates the axial electron density under attenuation conditions. In contrast to continuous propagation, the electron density is on the order of 10^19^ m^−3^, indicating a relatively weaker discharge state. The electron density initially increases and subsequently decreases, reaching a peak value of 7.26 × 10^19^ m^−3^ at 0.6 ns before gradually declining to 7.2 × 10^18^ m^−3^ at 1.7 ns. The spatial distribution of the electron density is presented in Figure 5(b1–b5).

**Figure 7:**
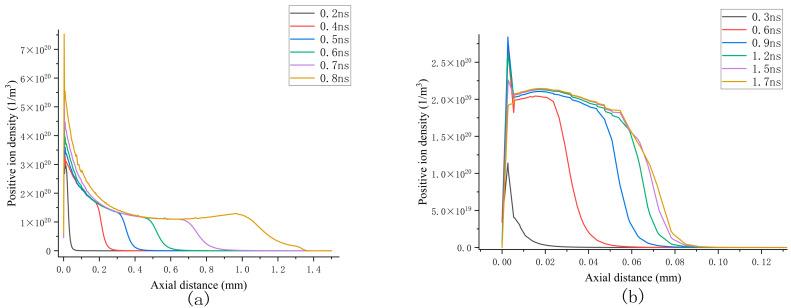
Axial distributions of positive ions under continuous and attenuation propagation conditions: (**a**) continuous transmission situation; (**b**) attenuation propagation situation.

**Figure 9:**
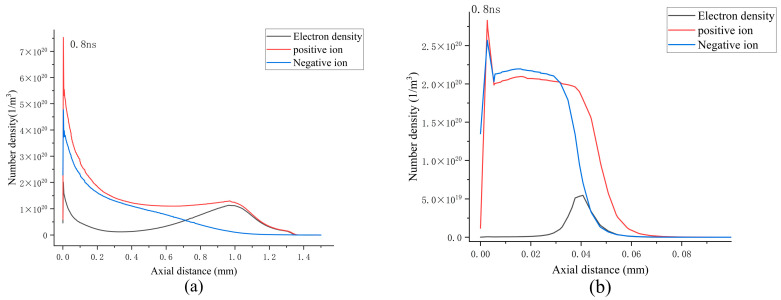
Axial particle distributions at 0.8 ns under continuous propagation and attenuation propagation conditions: (**a**) continuous transmission situation; (**b**) attenuation propagation situation.

Figure 7a illustrates the axial distribution of the positive ion density under continuous propagation conditions. Positive ions exhibit an aggregation effect near the particle tip and propagate towards the negative electrode along with the streamer under the influence of the applied electric field. Within the channel, the ion density demonstrates a gradual increase trend, while in the previously propagated regions, the ion density remains uniformly distributed at an order of magnitude of approximately 10^20^ m^−3^. The propagation speed of positive ions ranges from 6.7 × 10^5^ m/s to 1.2 × 10^6^ m/s. A comparison with Figure 9a reveals that the peak electron density coincides spatially with the positive ion density, and that their propagation speeds are of the same order of magnitude. This indicates that the streamer’s gradual propagation towards the grounded electrode is primarily dominated by positive ions.

In the attenuated propagation scenario, positive ions fail to form a pronounced peak in ion density, which remains on the order of 10^20^ m^−3^, consistent with the continuous propagation case. This suggests that the attenuation of the streamer cannot be ascribed solely to the reduction in the positive ion density. Furthermore, it is noteworthy that, as depicted in the three axial distributions of particles in Figure 9, irrespective of whether the proportion of the mixed gas varies or not, the peak of positive ions consistently precedes both the electron density and the positive ion density at the streamer head, thereby highlighting the critical role of positive ions during propagation.

**Figure 8:**
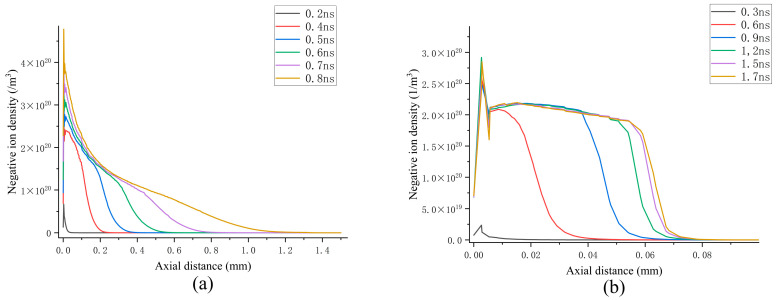
Axial distributions of negative ions under continuous and attenuation propagation conditions: (**a**) continuous transmission situation; (**b**) attenuation propagation situation.

Figure 8a illustrates the temporal evolution of the axial distribution of negative ions under continuous propagation conditions. As the streamer detaches from the particle tip and propagates towards the grounded electrode, negative ions are generated near the streamer head due to attachment reactions. The primary ions identified include SF_6_^−^, SF_5_^−^, SF_4_^−^, F_2_^−^, and others. Under the influence of the electric field, these negative ions migrate towards the particle tip, forming a peak. Furthermore, their number density exhibits a slight increase as the streamer extends. Over time, in regions far from the particle tip that have been traversed by the streamer, the density of negative ions is approximately one order of magnitude lower than that of positive ions and electrons. This further suggests that during the streamer propagation process, positive space charges play a dominant role in driving the propagation.

In the case of streamer attenuation propagation, the distribution patterns of negative ions and positive ions are consistent, with their number densities being on the same order of magnitude. However, a notable difference is that the concentration of negative ions is the highest among all the charged particles, which may account for the inability of the streamer to propagate continuously. Interestingly, the density of negative ions in the discharge channel remains largely unchanged across both propagation scenarios, suggesting that variations in the mixing ratios do not alter the electronegativity of the gas. Both SF_6_ and CF_4_ exhibit strong electronegativity, with comparable electron adsorption capabilities.

Figure 9 presents the axial distribution of each particle under continuous propagation conditions, providing a more intuitive comparison of the distributions of the three types of particles. The concentration of positive ions is the highest in the already-propagated channel and at the streamer head. At the particle tip, the density of positive ions constitutes a relatively large proportion among the charged particles and is on the same order of magnitude as that of electrons. This suggests that the ionization reaction may be intense throughout the process, particularly at the streamer head, where the densities of positive ions and electrons exceed that of negative ions, thereby controlling electron generation. This is likely to be the primary reason for the sustained development of the streamer.

Under attenuation conditions, the concentration of negative ions is the highest in the discharge channel. Within the streamer propagation channel, the electron density is one order of magnitude lower than that of both positive and negative ions. At the streamer head, the propagation of positive ions precedes that of negative ions and electrons, while the density of negative ions exceeds that of electrons by one order of magnitude. This suggests that negative ions play a dominant role in controlling the depletion of electrons throughout the entire propagation process.

**Figure 10:**
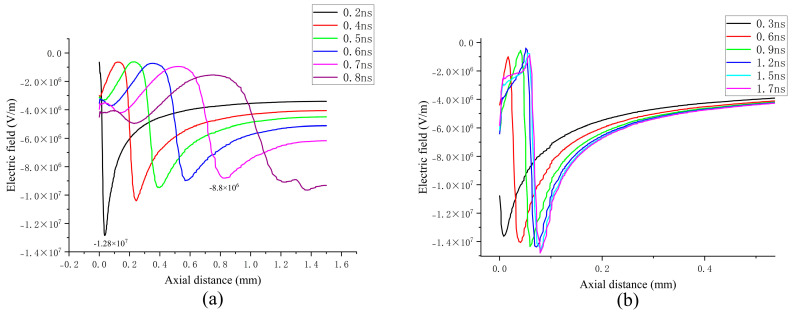
Axial electric field distributions under continuous and attenuation propagation conditions: (**a**) continuous transmission situation; (**b**) attenuation propagation situation.

The axial electric field distributions under both continuous propagation and attenuation propagation conditions are presented in Figure 10. As shown in the data of Figure 10a, during continuous propagation, the maximum electric field is located at the streamer head position, followed by a minimum electric field at subsequent spatial positions. Throughout the propagation process, the maximum electric field varies within the range of 1.28 × 10^7^ to 8.8 × 10^6^ V/m. In the streamer channel, the electric field decreases initially and subsequently increases from the tip to the streamer head, with variations spanning between 1 × 10^6^ and 2 × 10^6^ V/m.

As shown in Figure 10b, under the streamer attenuation condition, the maximum electric field along the axial direction of the streamer channel is one order of magnitude higher than that in the continuous propagation case and exhibits a gradually increasing trend. According to Morrow’s research [37,38], the primary mechanism for streamer attenuation in air or pure SF_6_ involves the continuous reduction in the electric field at the streamer head. However, in the SF_6_/CF_4_ mixed gas (with 40% CF_4_ content), an anomalous phenomenon was observed. During the gradual fading of the streamer, the electric field at the streamer head remained at a high level without any significant decrease, thereby excluding the possibility that the streamer attenuation was caused by the decay of the electric field.

**Figure 11:**
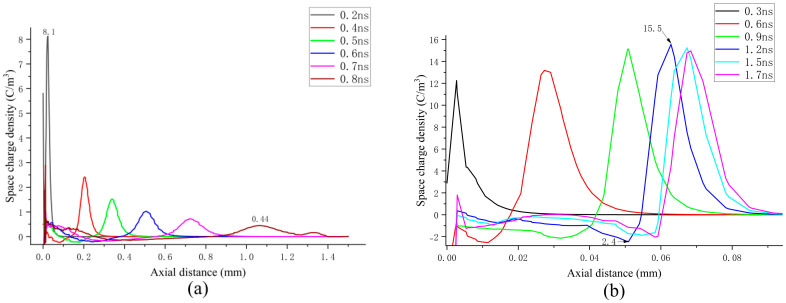
Axial space charge distributions under continuous propagation and attenuation propagation conditions: (**a**) continuous transmission situation; (**b**) attenuation propagation situation.

Figure 11a displays the axial spatial net charge density under continuous propagation conditions. It is evident that the highest positive charge density is located at the streamer head. As the streamer propagates toward the grounded electrode, the maximum positive charge density exhibits a decreasing trend, with values ranging from 0.44 to 8.1 C/m^3^. Figure 11b illustrates the axial spatial net charge density under attenuated propagation conditions. The peak of the positive spatial net charge initially increases and subsequently decreases, with the maximum value reaching approximately 15.5C/m^3^, which is significantly higher than that observed under continuous propagation conditions.

In the case of streamer attenuation, the variation trend of the positive space charge near the streamer head is in agreement with the trend of the electric field at the streamer head. Within the discharge channel, a peak in the space charge forms at the streamer head. Furthermore, a region of negative space charge is evident in proximity to the streamer head. In the continuous propagation scenario, the negative space charge exhibits minimal magnitude and uniform distribution. For attenuated propagation, the absolute value of the negative space charge density remains within 2.5 C/m^3^. When compared to the uniform distribution of the negative space charge during continuous propagation, the “negative charge center” emerges near the “positive charge center” at the streamer head during streamer attenuation at 1.2 ns, where the negative charge density reaches 2.4 C/m^3^. During streamer attenuation, as the maximum positive charge near the streamer head increases, the negative charge gradually accumulates at the negative charge center. Referring to Figure 6b, it can be observed that the decrease in the electron density coincides with the increase in the negative charge, suggesting that a strong attachment reaction may occur in the streamer channel, leading to the generation of negative ions as electrons are consumed. From the weakening effect associated with the accumulated negative charge, it can be inferred that the buildup of negative charge at the negative charge center might be essential in limiting the strong ionization area and facilitating streamer decay.

**Figure 12:**
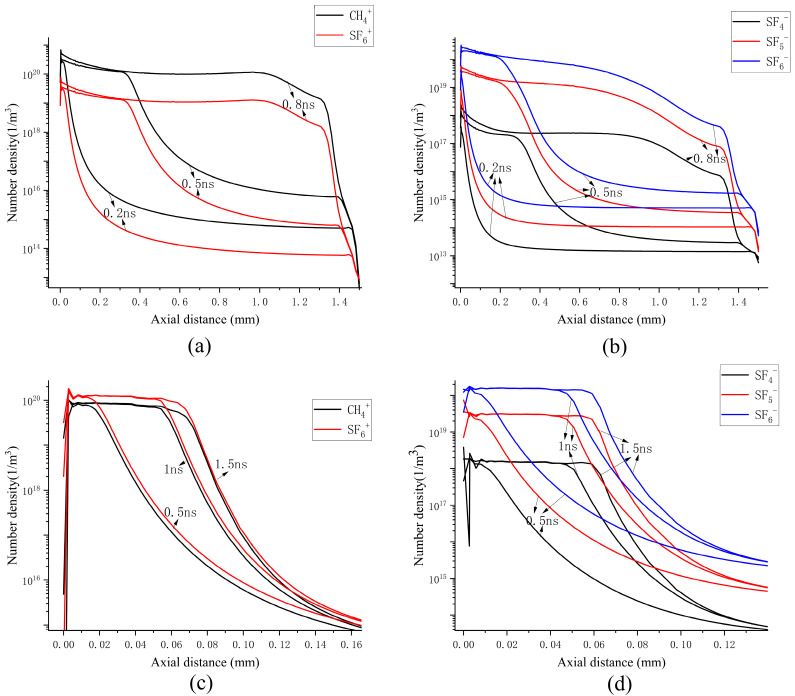
Axial number densities of various positive and negative ions under (**a**,**b**) continuous propagation conditions and (**c**,**d**) attenuation propagation conditions in SF_6_/CF_4_ mixtures.

**Figure 13:**
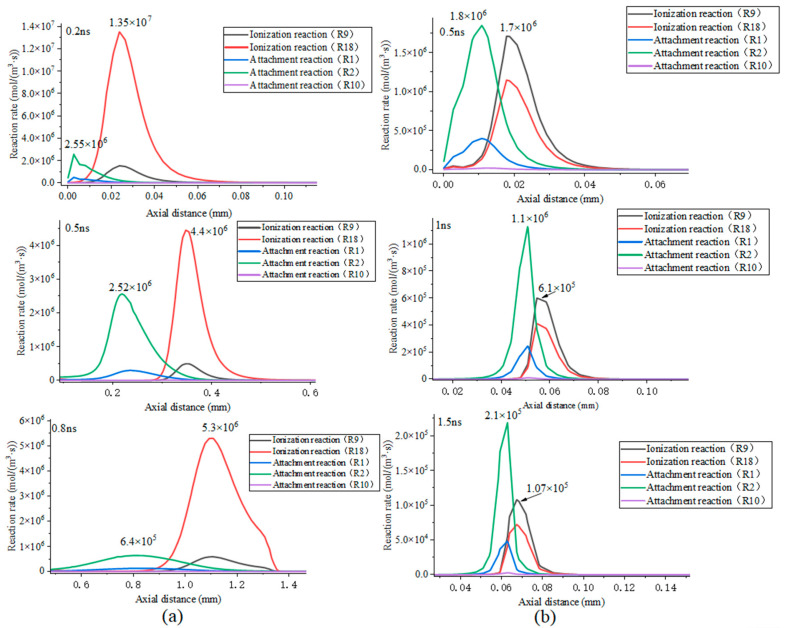
Axial distributions of ionization and attachment reaction rates under continuous and attenuated propagation conditions: (**a**) continuous transmission situation; (**b**) attenuation propagation situation.

The distribution of ion species along the axial direction under the two different concentration conditions are presented in Figure 12. In the positive space charge region at the streamer head, it is composed of CF_4_^+^ and SF_6_^+^. In both cases, the number densities of CF_4_^+^ and SF_6_^+^ at the streamer head range from 10^19^ m^−3^ to 10^20^ m^−3^. The difference arises from the varying proportions of positive ions due to the distinct ratios of the mixed gases. When the proportion of CF_4_ in the mixed gas is higher, the concentration of CF_4_^+^ is greater; conversely, when the proportion decreases, the concentration of CF_4_^+^ diminishes accordingly. The variation trends of the number densities of CF_4_^+^ and SF_6_^+^ are consistent with the corresponding trends of the spatial positive charge concentration and the electric field at the streamer head, suggesting that positive ions facilitate the propagation of streamers by enhancing the electric field at their heads. The ionization reaction rates of CF_4_^+^ and SF_6_^+^ are presented in Figure 13a. It can be observed that, when the concentration of CF_4_ is 90%, the ionization rate of SF_6_ (R9) at the streamer head is one order of magnitude lower than that of CF_4_ (R18), suggesting that during continuous streamer propagation, the ionization of CF_4_ serves as the primary mechanism for the generation of positive ions. In the case of streamer attenuation, the ionization reaction rates of CF_4_ and SF_6_ at the streamer head are one order of magnitude lower than those during continuous propagation. As shown in Figure 13b, the ionization rate of SF_6_ (R9) at the streamer head is higher than that of CF_4_ (R18), indicating that different mixing ratios alter the primary ionization reaction species at the streamer head, thereby influencing the ionization activity at the streamer head.

Negative ions play a critical role in determining whether propagation is continuous or attenuated. In the streamer channel, regardless of whether the propagation is continuous or attenuated, SF_5_^−^ and SF_6_^−^ are the predominant negative ions. Their number densities near the streamer head are more than one order of magnitude higher than that of SF_4_^−^, as illustrated in Figure 12b,d. Electrons are primarily generated at the streamer head, and at the location of the minimum ionization rate, the attachment rates for the formation of SF_6_^−^ (R2) and SF_5_^−^ (R1) from SF_6_ are maximized. Consequently, during their migration towards the anode, the electrons generated at the streamer head attach to SF_6_ and SF_5_ molecules, thereby forming a region of negative space charge. When the concentration of CF_4_ is 90%, the streamer propagates continuously. The attachment rates of SF_6_^−^, SF_5_^−^, and SF_4_^−^ from SF_6_ are lower than the ionization rates of CF_4_ and SF_6_, resulting in no significant formation of negative space charge, as illustrated in Figure 11a. During streamer attenuation, the attachment rate close to the streamer head is considerably greater than the ionization rate. This leads to a substantial buildup of negative ions around the streamer head and facilitates the establishment of a negative space charge center. It can be observed that at 1.2 ns, the charge density of the negative space charge center reaches 2 C/m^3^. This leads to a change in the electric field near the streamer head within the streamer channel, enhances the attachment rate, accelerates the depletion of electrons at the streamer head, and thereby suppresses the development of the streamer.

Figure 13a presents the reaction rates of all the reactions under continuous propagation conditions. It is evident that ionization reactions R9 and R18, as well as attachment reactions R1 and R2, are the predominant reactions in the discharge space. Based on the peak positions and the comparison of the reaction types listed in Table 1, it can be observed that the peaks of both attachment reactions and ionization reactions occur at the same spatial location. In this study, the positions of the two peaks are defined as the “ionization center” and the “attachment center”. The ionization center is spatially ahead, where the ionization reaction rate of CF_4_ is the highest, ranging from 4.4 × 10^6^ to 1.37 × 10^7^ mol/(m^3^·s). At the attachment center, the SF_6_ attachment reaction R2 exhibits the highest reaction rate, albeit lower than the ionization reaction rate, allowing the streamer to continue developing. When the concentration of CF_4_ is 40%, the dominant ionization reaction at the ionization center shifts to SF_6_, with an ionization reaction rate ranging from 1.07 × 10^5^ to 1.7 × 10^6^ mol/(m^3^·s), which is one order of magnitude lower than that in the continuous propagation case. This further demonstrates that different mixing ratios significantly influence the ionization activity of the mixed gas. However, the primary reaction at the attachment center remains R2, with a reaction rate of approximately 2.1 × 10^5^ to 1.8 × 10^6^ mol/(m^3^·s). This indicates that variations in the concentration of CF_4_ affect the ionization intensity of the mixed gas by altering the main ionization reaction species. In the attenuation scenario, the attachment reaction rate exceeds the ionization reaction rate, leading to a greater disappearance of electrons compared to their generation, thereby causing the streamer to tend toward attenuation.

## 4. Conclusions

Based on the two-dimensional plasma fluid model, this study investigates the partial discharge phenomenon of free metal particles in SF_6_/CF_4_ mixed gas and systematically analyzes the spatiotemporal evolution characteristics of key parameters, including the electron density, positive and negative ion distributions, and axial electric field profiles, under various mixing ratios.

(1) The variation in the composition ratio of the mixed gas significantly influences the distribution characteristics of particle species within the discharge plasma. When the CF_4_ concentration is relatively high, positive ions dominate as the primary charged particles in the discharge space, particularly at the streamer head, where the number density of positive ions exceeds that of negative ions by approximately one order of magnitude. This results in the formation of a positive space charge region, which governs electron generation via field ionization and consequently facilitates the continuous propagation of the streamer channel.
(2) When the CF_4_ concentration is relatively low, negative ions dominate as the primary charged particles in the discharge space and establish a negative space charge region near the streamer head, thereby changing the local electric field. The electron attachment effect significantly enhances the recombination loss process, leading to the substantial suppression of streamer development and resulting in an attenuation characteristic.
(3) CF_4_ contributes to the dissipation of negative space charges and enhances the ionization activity of the mixed gas. By analyzing the distribution of space charges and the reaction rates, it is found that when the concentration of CF_4_ in the mixed gas reaches 90%, the ionization reaction rate increases by one order of magnitude compared to the low-concentration scenario (40% CF_4_). Moreover, near the streamer head, a negative space charge zone does not form as it does in the low-concentration CF_4_ case; instead, the charges are uniformly distributed throughout the discharge channel.

## Figures and Tables

**Table 1:** Chemical reaction Equations in SF_6_/CF_4_ mixtures.

Serial Number	Equation	Reaction Type
R1	e + SF_6_=>SF_5_^−^ + F	Attachment
R2	e + SF_6_=>SF_6_^−^	Attachment
R3	e + SF_6_=>SF_6_^−^	Attachment
R4	e + SF_6_=>e + SF_6_	Elastic collision
R5	e + SF_6_=>e + SF_6_(V1)	Vibrational excitation
R6	e + SF_6_=>e + SF_6_*	Excitation
R7	e + SF_6_=>e + SF_6_*	Excitation
R8	e + SF_6_=>e + SF_6_*	Excitation
R9	e + SF_6_=>2e + SF_6_^+^	Ionization
R10	e + SF_6_=>SF_4_^−^ + F_2_	Attachment
R11	e + SF_6_=>F_2_^−^ + SF_4_	Attachment
R12	e + CF_4_=>CF_2_^−^ + F_2_	Attachment
R13	e + CF_4_=>e + CF_4_	Elastic collision
R14	e + CF_4_=>e + CF_4_(V1)	Vibrational excitation
R15	e + CF_4_=>e + CF_4_(V2)	Vibrational excitation
R16	e + CF_4_=>e + CF_4_(V3)	Vibrational excitation
R17	e + CF_4_=>e + CF_4_*	Excitation
R18	e + CF_4_=>2e + CF_4_^+^	Ionization

## Data Availability

The data in this article are stated in the manuscript, do not conflict with other articles, and can be published publicly.
